# Analysis of nursing practice environment and its influencing factors: a cross-sectional study in a maternal and child specialized hospital in China

**DOI:** 10.3389/frhs.2026.1922759

**Published:** 2026-10-22

**Authors:** Fang Wu, Huimin Li, Shaochuan Chen

**Affiliations:** Nursing Department, School of Medicine, Chengdu Women’s and Children’s Central Hospital, University of Electronic Science and Technology of China, Chengdu, Sichuan, China

**Keywords:** influencing factors, maternal and child specialized hospital, nurses, occupational health, practice environment

## Abstract

**Objective:**

This study aims to evaluate the current status of the nursing practice environment in a maternal and child specialized hospital, identify its associated influencing factors, and provide an empirical reference for formulating targeted optimization strategies for the nursing practice environment.

**Methods:**

A questionnaire survey was conducted using the Chinese version of the Nursing Practice Environment Scale among 1,154 nurses working at a tertiary maternal and child specialized hospital in Chengdu.

**Results:**

The total mean score of the nursing practice environment in the investigated maternal and child specialized hospital was (78.46 ± 14.67). The highest domain score was for quality management (89.52 ± 14.07), while the lowest was for participation in hospital management (59.65 ± 29.28). Department, gender, and professional position were identified as influencing factors of the nursing practice environment (*P* < 0.05).

**Conclusion:**

The score of the nursing practice environment in the maternal and child specialized hospital in Chengdu was lower than that in similar hospitals in coastal regions, indicating a need for further improvement. Administrators should focus on addressing the differentiated needs of nurses working in emergency departments and intensive care units, male nurses, and staff nurses. Effective mechanisms should be established to enhance nurses’ participation in hospital management, thereby strengthening their sense of professional belonging. Continuous optimization of the practice environment is essential to promote nursing workforce stability and improve the quality of nursing care.

## 1 Introduction

The nursing practice environment refers to the organizational factors in the workplace that promote or constrain professional nursing practice, such as the degree of nurses’ participation in hospital management, organizational support for nursing practice, nursing leadership, nurse staffing, professional development opportunities for nurses, nurse compensation, nurse-physician collaboration, and the social status of nurses (1). The practice environment encompasses both the physical and social settings in which nurses work, and these factors directly influence nurses’ job performance, professional satisfaction, and the quality of patient care. A healthy nursing practice environment provides adequate resources, support, and a positive work climate, thereby enhancing nurses’ job satisfaction and the quality of patient care (2, 3). Research has shown that improving the nursing practice environment can significantly reduce nurses’ work-related stress and burnout, thereby increasing their intention to stay and work efficiency (4, 5).

This study employs the Job Demands-Resources (JD-R) model as its theoretical framework, conceptualizing the practice environment as comprising two categories of elements: job demands and job resources. The JD-R model posits that job demands (e.g., high workload, emotional exhaustion) deplete individuals’ physical and psychological resources, whereas job resources (e.g., organizational support, leadership, and career development opportunities) provide buffering and motivational functions, with the dynamic balance between the two determining nurses’ work engagement and burnout levels (6, 7). This model has been widely applied in nursing research. For instance, a study by Wang et al., grounded in the JD-R model, demonstrated that the practice environment exerts a significant negative effect on nurses’ work stress (*β*=−0.337) and indirectly influences patient safety attitudes through work stress (8). Meanwhile, organizational support theory provides another important perspective for this study: hospitals’ valuation of and investment in nurses enhance nurses’ organizational commitment and work engagement by satisfying their socioemotional needs (respect, recognition, belonging) and instrumental needs (resources, information, support). A cross-sectional study by Zheng et al. involving 792 nurses confirmed that perceived organizational support has a direct positive effect on nurses’ occupational well-being (*β* = 0.323), with quality of working life and decent work perception serving as sequential mediators (9, 10).

As core institutions safeguarding the health of women and children, maternal and child specialized hospitals have a nursing practice environment that directly affects the quality of care, patient safety, and nurses’ professional development. Nurses in maternal and child specialized hospitals face greater pressure compared with those in other types of hospitals, primarily due to the higher workload and more complex patient needs inherent in maternal and child nursing care (11, 12). Nurses in maternal and child specialized hospitals face three fold superimposed pressures: first, scenarios such as obstetric emergencies and neonatal resuscitation demand rapid responses, while situations including perinatal care and mother-infant separation involve high-intensity emotional investment; second, patient safety requirements are characterized by a “zero-tolerance” specificity, with maternal and infant safety incidents attracting far greater public attention and accountability pressure than general departments, keeping nurses in a state of chronic hyper-vigilance; third, specialized nursing competency requirements are becoming increasingly refined, as technological advances in assisted reproduction and high-risk pregnancy management pose continuous challenges to nurses’ knowledge renewal capacity.

This high-intensity work environment may lead to nurse burnout and decreased job satisfaction, thereby compromising the quality of care and patient safety (5, 13). Therefore, improving the nursing practice environment in maternal and child specialized hospitals is of great importance. Such improvements not only enhance nurses’ job satisfaction and intention to stay but also improve the quality and safety of patient care (14, 15).

Therefore, to investigate the current state of the nursing practice environment in maternal and child specialized hospitals, a survey on the practice environment was conducted among nurses in such hospitals. This study aims to provide an evidence-based foundation for the scientific development of improvement strategies for the nursing practice environment. The report is presented as follows.

## 2 Methods

### 2.1 Study design

This study employed a cross-sectional design, utilizing a self-administered online questionnaire for data collection.

This study was designed and reported in accordance with the Strengthening the Reporting of Observational Studies in Epidemiology (STROBE) checklist for cross-sectional studies. A cluster sampling method was employed, and the survey was conducted via the National Nursing Quality Data Platform between November and December 2024 in a maternal and child specialized hospital. The hospital is a tertiary maternal and child health institution, responsible for regional maternal and child healthcare services. It comprises clinical departments, including obstetrics, gynecology, pediatrics, neonatology, emergency department, and the intensive care unit (ICU), with 1,733 open beds and approximately 14,000 annual deliveries.

The nursing work in this hospital exhibits typical characteristics of maternal and child specialty care, including high-tempo work scenarios such as obstetric emergencies and neonatal resuscitation, high-emotional-investment situations such as perinatal care and mother-infant separation, as well as heightened accountability pressure and risk sensitivity arising from maternal and infant safety incidents. The contradiction between job demands and resource supply in the nurses’ practice environment is particularly prominent.

### 2.2 Participants

#### 2.2.1 Target population and accessible population

The target population comprised all nursing staff at the hospital who met the inclusion criteria. The accessible population comprised nurses who were on duty during the survey period, provided informed consent, and voluntarily participated in the survey.

#### 2.2.2 Cluster definition and selection

Departments (nursing units) served as the cluster units for cluster sampling. The hospital comprises clinical departments, including obstetrics, gynecology, pediatrics, neonatology, the emergency department, and the intensive care unit (ICU). All nursing units across the hospital were included as clusters, encompassing a total of 49 nursing units and covering 1,377 nurses in nursing positions. Since the study aimed to assess the current practice environment of all nurses in the hospital, no random sampling of clusters was performed; instead, all nursing units were included as clusters.

#### 2.2.3 Participant screening procedure

The nursing department distributed the questionnaire uniformly through the National Nursing Quality Data Platform, and head nurses in each department organized nurses in their respective units to complete it. Nurses read the informed consent statement before completing the questionnaire and participated voluntarily. After submission, the researchers reviewed the questionnaires and excluded those with incomplete information or patterned responding.

### 2.3 Inclusion and exclusion criteria

#### 2.3.1 Inclusion criteria

Holding a valid nurse practice certificate; having worked in a nursing position for ≥50% of the time during the current year; voluntary participation in the study.

#### 2.3.2 Exclusion criteria

Nurses with less than one year of tenure; nurses in non-nursing positions.

#### 2.3.3 Sample size calculation

Based on the sample size formula for estimating a population mean in cross-sectional surveys, and referencing the standard deviation of the nurse practice environment assessment score reported in previous literature (16) (*σ* = 14.67), with a permissible error of *δ* = 4.05 (5% of the mean) and a confidence level of 95% (Z = 1.96), the minimum required effective sample size was calculated to be approximately 53. This study ultimately obtained 1,154 valid questionnaires, far exceeding the minimum sample size requirement.

#### 2.3.4 Statistical assumptions

Sample size estimation was performed at a 95% confidence level with a permissible error of 5% of the mean; the valid questionnaire response rate was assumed to be no less than 95%; two-sided tests were used, with *P* < 0.05 considered statistically significant.

### 2.4 Research tools

#### 2.4.1 General information questionnaire

This included nurse gender, age, educational level, department, professional title, position, and whether they were a permanent staff member (i.e.,), employed in a state-funded position).

#### 2.4.2 Nursing practice environment scale

The Nursing Practice Environment Scale was developed under the leadership of the Nursing Center of the Hospital Management Institute, National Health Commission of the People's Republic of China. Constructed on the basis of the Chinese version of the Practice Environment Scale of the Nursing Work Index and the Nursing Practice Environment Scale established by international scholars, this instrument was adapted to the national context of China via systematic literature review, followed by revisions after three rounds of expert consultation and a preliminary pilot study.

The scale consists of 37 items categorized into 10 dimensions, specifically: participation in hospital management (items 1–3), clinical nursing professionalism (items 4–5), leadership and communication (items 6–9), quality management (items 10–15), internal support (items 16–20), nurse-physician collaboration (items 21–22), professional development (items 23–26), staffing and resource adequacy (items 27–31), social status (items 32–33), and compensation (items 34–36), with item 37 designed as an overall evaluation item. This is a self-report scale, where each item is scored on a 0–100 numeric rating scale, with 0 representing “strongly disagree” and 100 representing “strongly agree.” Participants rate each item in accordance with their personal perceptions to assess the nursing practice environment of their affiliated hospital. The average total score is obtained by summing the scores of all items and dividing by the total number of items, with higher scores indicating a more favorable nursing practice environment. Previous psychometric tests have shown that the Cronbach's *α* coefficient of the scale is 0.985, and the cumulative variance contribution rate for construct validity reaches 87.576%, demonstrating that the scale has good reliability and validity (17).

### 2.5 Ethical considerations

The anonymity and voluntary nature of participation were rigorously safeguarded throughout this study. The first page of the questionnaire featured an informed consent statement that clearly outlined the research purpose and methodology, described the potential risks and benefits for participating nurses, and indicated that completion of the questionnaire would require approximately five minutes. Participants were explicitly advised that they could discontinue their involvement at any point without facing any unfavorable repercussions. All response data were used exclusively for academic research and were stored with password-restricted access, available only to designated members of the research team. The study protocol received formal approval from the Ethics Committee of Chengdu Women's and Children's Central Hospital (Ethics Approval No. 2024[18]), and the entire investigation was carried out in full compliance with the principles set forth in the World Medical Association's Declaration of Helsinki.

### 2.6 Data collection

This online survey was administered via the Questionnaire Star platform. A total of 1,377 nurses were employed in nursing positions at the target hospital and were eligible for participation. A total of 1,213 nurses completed the survey, yielding a response rate of 88.09%. Following questionnaire collection, the research team reviewed all responses and excluded invalid questionnaires based on predefined criteria, including incomplete answers and patterned response styles (e.g., selecting identical extreme options across all items). Ultimately, 1,154 valid questionnaires were retained, resulting in an effective response rate of 95.14%.

### 2.7 Data analysis

SPSS 27.0 was used for data entry and analysis. Continuous variables were described as the mean ± standard deviation, and categorical variables as frequencies and percentages. Differences in total scores of the Nursing Practice Environment Scale across demographic groups were compared using one-way ANOVA and independent-samples t-tests. Multiple linear regression was applied to identify influencing factors of the nursing practice environment. Statistical significance was set at *P* < 0.05.

## 3 Results

### 3.1 General information

A total of 1,154 nurses were enrolled in this study. The mean age of the participants was 35.17 ± 7.77 years. Among them, 1,133 (98.18%) were female, and 21 (1.82%) were male. The majority of nurses had more than five years of work experience (67.59%). Nurses holding the title of supervisor or above accounted for 54.33% of the sample. Frontline nurses comprised 88.39% of the participants, and the majority held a bachelor's degree or higher (80.33%). Nurses without permanent hospital employment status (i.e., contract, or temporary staff) accounted for 83.62% of the sample, see Table 1 for details.

**Table 1:** General characteristics of the study participants (*n* = 1,154).

Variable	Category	*n* (%)	Practice environment score (mean ± SD)	t/*F*	*P*
Age	≤25	23 (2.0)	77.27 ± 14.66	0.679	0.607
	26–30	389 (33.7)	77.82 ± 15.68		
	31–35	279 (24.2)	78.33 ± 14.71		
	36–40	221 (19.2)	78.53 ± 14.96		
	≥41	242 (20.9)	79.72 ± 12.57		
Gender	Male	21 (1.8)	68.53 ± 16.83	3.144	0.002
	Female	1,133 (98.2)	78.65 ± 14.57		
Education level	Secondary Vocational School	9 (0.8)	81.41 ± 12.93	0.493	0.687
	Associate degree	194 (16.8)	77.80 ± 14.34		
	Bachelor's degree	927 (80.3)	78.51 ± 14.82		
	Master's degree or above	24 (2.1)	81.00 ± 12.43		
Professional title	Nurse	39 (3.4)	77.67 ± 13.85	1.199	0.309
	Senior nurse	488 (42.3)	77.96 ± 15.38		
	Supervisor nurse	504 (43.7)	78.46 ± 14.67		
	Associate Chief Nurse or above	123 (10.6)	80.72 ± 11.67		
Position	Staff nurse	1,096 (95.0)	78.13 ± 14.85	4.071	0.007
	Head nurse	55 (4.7)	85.05 ± 8.76		
	Chief Head Nurse	1 (0.1)	88.17 ± 0.00		
	Nursing Department Director	2 (0.2)	78.31 ± 2.24		
Department	Obstetrics and Gynecology	307 (26.6)	81.99 ± 11.24	10.978	<0.001
	Pediatrics	306 (26.5)	79.30 ± 15.26		
	Operating room	90 (7.8)	77.73 ± 15.30		
	Outpatient department	161 (14.0)	80.14 ± 14.14		
	Emergency department	62 (5.4)	72.09 ± 17.07		
	Intensive Care Unit (ICU)	193 (16.7)	72.71 ± 15.89		
	Others	35 (3.0)	77.42 ± 13.34		
Employment status	Contract	965 (83.6)	78.03 ± 15.12	2.299	0.022
	Permanent	189 (16.4)	80.70 ± 11.91		
Years of work experience	≤1 year	9 (0.8)	77.79 ± 14.84	2.879	0.022
	2–4 years	154 (13.3)	80.25 ± 14.21		
	5–9 years	328 (28.4)	76.25 ± 16.24		
	10–19 years	452 (39.2)	79.32 ± 14.26		
	≥20 years	211 (18.3)	78.80 ± 12.94		

### 3.2 Scores of nursing practice environment

The mean total score on the Nursing Practice Environment Scale in the maternal and child specialized hospital was 78.46 ± 14.67. Among the six dimensions, quality management scored the highest, while participation in hospital management scored the lowest. The top five items were predominantly related to hospital training systems and workflow standardization, whereas the bottom five items were mainly concentrated in the areas of hospital management participation and salary equity, details are shown in Tables 2–4.

**Table 2:** Dimension scores of the nursing practice environment (*n* = 1,154).

Dimension	Number of items	Item-level score (mean ± SD)
Quality management	6	89.52 ± 14.07
Professional development	4	88.09 ± 17.33
Staffing and resource adequacy	5	87.47 ± 15.56
Nurse-physician collaboration	2	87.07 ± 17.33
Clinical nursing professionalism	2	86.96 ± 17.71
Internal support	5	84.10 ± 17.12
Leadership and communication	4	83.97 ± 19.32
Social status	2	78.19 ± 15.56
Compensation	3	78.15 ± 22.37
Participation in hospital management	3	59.65 ± 29.28

**Table 3:** The top 5 highest-scoring items on the nursing practice environment scale (*n* = 1,154).

Item No.	Item	Score (mean ± SD)
20	The hospital has a clear post-exposure management process for occupational exposure and implements it effectively	93.58 ± 13.90
23	The hospital provides systematic training for newly recruited nurses	93.35 ± 13.92
28	Nurses on the work team are competent to perform nursing duties	91.44 ± 15.22
11	Work systems and protocols are well established	90.99 ± 14.87
24	The hospital provides continuing education for nurses based on job requirements	90.52 ± 16.26

**Table 4:** Bottom 5 items on the nursing practice environment scale (*n* = 1,154).

Item No.	Item	Score (mean ± SD)
2	Nurses have opportunities to decide on hospital affairs	54.23 ± 32.21
1	Nurses have opportunities to participate in hospital internal management	62.17 ± 31.02
3	Nurses have opportunities to serve as members of hospital management-related committees	62.55 ± 31.56
35	Nurses’ compensation is at a reasonable level compared with other occupations in society	75.26 ± 25.01
17	Hospital administrative departments can support nursing work	76.16 ± 26.11

### 3.3 Multiple linear regression analysis of the nursing practice environment

#### 3.3.1 Results of univariate analysis

Univariate analysis showed that department, gender, position, employment status, and years of experience were significantly associated with the nursing practice environment (*P* < 0.05).

#### 3.3.2 Results of multiple regression analysis

Taking the score of the nursing practice environment as the dependent variable, the statistically significant variables from the univariate analysis were entered as independent variables into a multiple linear regression model. Among these, multicategorical variables were dummy-coded, with the specific assignment method shown in Table 5. The regression analysis revealed that department, gender, and position were the main factors influencing the nursing practice environment (*P* < 0.05), as detailed in Table 6.

**Table 5:** Variable assignment for multiple linear regression analysis.

Variable	Assignment	Variable	Assignment
Department		Years of work experience	
Obstetrics and Gynecology	(Yes = 1, No = 0)	≤1 year	(Yes = 1, No = 0)
Pediatrics	(Yes = 1, No = 0)	2–4 years	(Yes = 1, No = 0)
Operating room	(Yes = 1, No = 0)	5–9 years	(Yes = 1, No = 0)
Outpatient department	(Yes = 1, No = 0)	10–19 years	(Yes = 1, No = 0)
Emergency department	(Yes = 1, No = 0)	≥20 years	(Yes = 1, No = 0)
Intensive care unit (ICU)	(Yes = 1, No = 0)	Employment status	
Others	(Yes = 1, No = 0)	Contract	(Yes = 1, No = 0)
Position		Permanent	(Yes = 1, No = 0)
Staff nurse	(Yes = 1, No = 0)	Gender	
Head nurse	(Yes = 1, No = 0)	Male	(Yes = 1, No = 0)
Chief head nurse	(Yes = 1, No = 0)	Female	(Yes = 1, No = 0)
Nursing department director	(Yes = 1, No = 0)		

**Table 6:** Multiple linear regression analysis of factors influencing the nursing practice environment.

Independent Variables	β	SE	β′	*t*	*P*
(Constant)	81.639	0.821		99.435	<0.001
Male	−8.272	3.162	−0.075	−2.616	0.009
Department					
Emergency department	−9.577	1.983	−0.014	−4.83	<0.001
Intensive care unit (ICU)	−8.906	1.31	−0.227	−6.799	<0.001
Position					
Head nurse	6.141	1.971	0.089	3.116	0.002

R = 0.262, R^2^ = 0.068, adjusted R^2^ = 0.060, F = 8.390, *P* < 0.001.

## 4 Discussion

### 4.1 Analysis of the current state of the nursing practice environment in maternal and child specialized hospitals

The mean total score on the Nursing Practice Environment Scale in the maternal and child specialized hospital was 78.46 ± 14.67, which was higher than that reported for nurses in 21 tertiary hospitals in Sichuan Province (70.85 ± 18.69) (18), but slightly lower than that reported for nurses in a tertiary maternal and child specialized hospital in Guangzhou (80.99 ± 14.67) (16). This finding suggests that the nursing practice environment in this hospital is acceptable but still has room for improvement.

Several factors may account for this. First, the hospital has closely followed the national “14th Five-Year Plan” for nursing development and the Action Plan for Further Improving Nursing Services (2023–2025), creating favorable conditions for nurse training and professional development over the past two years. Second, the hospital has improved its incentive mechanisms and implemented a scientific nurse evaluation and promotion system. Third, the hospital has taken practical measures to reduce the nurse workload, including the phased rollout of mobile nursing PDAs, the employment of medical nursing assistants, and the implementation of “Internet + nursing services,” all of which have improved nursing efficiency and enhanced nurses’ sense of professional fulfillment. Nevertheless, compared with maternal and child specialized hospitals in coastal regions, gaps may still exist in staffing, nursing management approaches, and career development support. Future efforts should focus on strengthening nurse staffing, promoting empowered nursing management, and improving career development and research support systems to comprehensively enhance the quality of the nursing practice environment.

Among the specific dimensions, quality management received the highest score, likely due to the hospital's well-established quality standards, evaluation indicators, monitoring mechanisms, and closed-loop improvement processes. This study also found that items such as “The hospital has a clear post-exposure management process for occupational exposure and implements it effectively,” “The hospital provides systematic training for newly recruited nurses,” “Nurses in the work team are competent to perform nursing duties,” “Work systems and protocols are well established,” and “The hospital provides continuing education for nurses based on job requirements” scored relatively high. These findings indicate that the hospital has achieved notable success in hospital infection management, the development of nursing management systems, the implementation of standardized training, and the assurance of job competence. These initiatives have strengthened the foundation of quality management, clarified nursing role responsibilities, and established operational systems and procedures. The nursing team has fostered a positive atmosphere of proactively identifying problems and collaboratively implementing improvements, which has significantly enhanced nurses’ recognition of the quality management dimension.

The three lowest-scoring items were “Nurses have opportunities to decide on hospital affairs,” “Nurses have opportunities to participate in hospital internal management,” and “Nurses have opportunities to serve as members of hospital management-related committees.” This indicates that nurses in the maternal and child specialized hospital perceived limited opportunities to participate in hospital affairs and management. These findings are consistent with those of Zhang et al. (2). Such limited participation not only affects nurses’ professional development but may also negatively impact the overall management efficiency of the hospital.

From the perspective of employee empowerment theory, empowerment comprises two dimensions: “structural empowerment” and “psychological empowerment."The former refers to employees’ access to resources, support, information, and development opportunities, while the latter refers to employees’ perceived autonomy, competence, work meaning, and impact (19). The hospital has established an institutional foundation at the structural empowerment level; however, nurses’ low perception of “participation in hospital affairs” indicates that structural empowerment has not yet been effectively translated into psychological empowerment.

When nurses perceive insufficient managerial support, they often remain silent due to feelings of powerlessness, which further restricts their participation in hospital affairs (20). This phenomenon may weaken nurses’ sense of professional belonging and ownership and, to some extent, limits the integration of nursing perspectives into hospital management decisions. It is recommended that administrators expand channels for nurses to participate in hospital affairs, such as including nurses in hospital-level decision-making bodies and establishing a nursing proposal feedback system, to effectively enhance nurses’ presence and influence in management decisions.

The relatively low scores on the items “Nurses’ compensation is at a reasonable level compared with other occupations in society” and “Hospital administrative departments can support nursing work” suggest that nurses in the maternal and child specialized hospital are dissatisfied with their compensation levels, and that administrative support for nursing work requires further strengthening. Research has shown that nurses’ work environment and compensation levels directly affect their job satisfaction and intention to stay (21). Organizational support has been found to mitigate the negative effects of patient-related stress on nurse emotional exhaustion (22). From the perspective of organizational support theory, pay satisfaction and perceived administrative support serve as core signals by which nurses judge whether “the organization values their contributions and cares about their well-being” (23, 24). When scores on these two dimensions are low, nurses’ overall perception of organizational support is weakened, thereby affecting their affective commitment and work engagement. Reasonable compensation and effective administrative support are key factors in ensuring a stable and efficient nursing workforce. By combining financial incentives with organizational support, hospitals can improve both nurse job satisfaction and overall quality of care.

### 4.2 Analysis of factors influencing the nursing practice environment in maternal and child specialized hospitals

#### 4.2.1 Department

The results of this study indicate that practice environment scores among emergency department and ICU nurses were significantly lower than those in obstetrics, gynecology, pediatrics, and outpatient departments. This may be related to the more severe occupational stress, higher workload, and more complex clinical situations faced by nurses in these two departments. According to a study of emergency department nurses in New Jersey, 72% of registered nurses reported moderate-to-complete burnout, while 34% reported persistent or complete burnout, with high workload and an unsupportive practice environment significantly increasing the likelihood of burnout (4). ICU nurses likewise face a high-intensity work environment, where emotional exhaustion, depersonalization, and reduced personal accomplishment are more prevalent (25). In contrast, work environments in departments such as obstetrics, gynecology, pediatrics, and outpatient clinics are relatively more supportive, with nurses experiencing comparatively lower workloads, which contributes to improved nursing quality and patient safety (26).

The lower practice environment scores among emergency department and ICU nurses can be interpreted through the lens of employee empowerment theory. Empowerment theory emphasizes that employees’ positive perceptions of the work environment stem from their attainment of work meaning, competence, autonomy, and impact (27). When nurses are chronically exposed to high-demand, low-control work situations, their access to resources, support, information, and development opportunities is constrained, resulting in insufficient structural empowerment, which in turn weakens positive evaluations of the practice environment. Therefore, the lower practice environment scores among emergency department and ICU nurses essentially reflect a structural deficiency in organizational empowerment mechanisms rather than inadequate individual adaptation. Nursing managers can enhance structural empowerment by optimizing staffing allocation, establishing flexible scheduling, and strengthening nurses’ sense of control over their work pace.

#### 4.2.2 Gender

The results of this study indicate that female nurses scored higher on the practice environment than male nurses, consistent with the findings of Wang Tiemei et al. (18). A systematic review on the mental health and well-being of male nurses noted that male nurses face significant cultural and stereotypical barriers in nursing practice, which can generate feelings of exclusion, loneliness, and a lack of professional identity, thereby serving as precursors to more profound mental health problems (28). In a nursing workforce overwhelmingly dominated by women, male nurses may encounter difficulties in social integration due to the absence of a homogeneous peer group. Qualitative research has shown that male and female nurses experience different advantages and disadvantages in the workplace, involving two core themes: the influence of gender on work practice and the gendered image of nursing and communication (29). Moreover, when providing care involving patients’ intimate body parts, male nurses frequently encounter distrust or even hostility from patients and their families, leading to anxiety, embarrassment, or tension that affects their professional experience and psychological well-being (28).

Previous studies have demonstrated that perceived organizational support indirectly influences turnover intention through “meaning in work.” The implicit exclusion experienced by male nurses in maternal and child specialty care settings may challenge their construction of meaning in nursing work, thereby lowering their evaluation of the practice environment (30). Enhancing male nurses’ perception of the practice environment requires not only increasing supportive resources but also helping them reaffirm the professional value of nursing work in gender-sensitive contexts.

#### 4.2.3 Position

The results of this study indicate that position is an influencing factor in the assessment of the nursing practice environment, with staff nurses scoring significantly lower than head nurses. This finding is consistent with that of He et al. (31). Head nurses, as nursing managers, participate in hospital management decision-making, resource allocation, and scheduling, and possess a certain degree of voice and autonomy in policy implementation and process optimization; their perception of the practice environment focuses more on overall management effectiveness. Head nurses also enjoy greater organizational resources in terms of information access, learning opportunities, and awards and promotions, and have a relatively flexible work pace and a certain buffer space in their daily work.

From the perspective of shared governance theory, the perceptual gap between staff nurses and head nurses is essentially a manifestation of unequal distribution of decision-making participation rights. Shared governance can enhance nurses’ sense of empowerment and autonomy, and improve job satisfaction and patient outcomes (32). In this study, “participation in hospital affairs” was the lowest-scoring item, corroborating the insufficient structural authorization of staff nurses. Many hospitals currently exhibit “formalistic embeddedness” in shared governance platforms have been established, yet nurses find it difficult to perceive their relevance to their own work (33). Therefore, the implication for nursing managers is that the key lies not in establishing additional governance platforms, but in ensuring that platforms possess “perceivable efficacy”, nurses’ suggestions can produce visible changes. A closed-loop feedback mechanism for nursing proposals should be established to transform “formalistic participation” into “substantive empowerment.”

In this study, multiple linear regression analysis showed that the model coefficient of determination was R^2^ = 0.068, with an adjusted R^2^ = 0.060 (F = 8.390, *P* < 0.001), indicating that the included demographic variables (gender, department, and position) explained only approximately 6.0% of the total variance in nurse practice environment scores. Although the regression model was statistically significant overall, with gender, emergency/ICU work background, and head nurse position reaching statistical significance in the model, the low R^2^ value indicates that the predictive power of the model is limited. This finding is consistent with previous studies concluding that the practice environment is influenced by multidimensional factors (34). Demographic variables can only statically describe nurses’ sociological characteristics and struggle to capture their dynamic experiences in the workplace (35). The limited explanatory power of the model suggests that the vast majority of variance in nurses’ perceptions of the practice environment may be determined by other factors not included in this model, including psychosocial variables such as workload, burnout, psychological safety, organizational culture, leadership style, and perceived organizational support. Future research should incorporate these core variables to construct a more comprehensive predictive model, thereby more accurately identifying the key factors influencing the nurse practice environment.

### 4.3 Limitations

This study has the following limitations. First, it was conducted at only one maternal and child specialized hospital, representing a single-center study with a homogeneous sample source. The findings of this study may be confounded by regional economic level, hospital grade, and specialty characteristics, resulting in limited external validity. Future research should prioritize multicenter designs, selecting maternal and child health institutions or general hospitals at various levels across provinces with different economic conditions in eastern, central, and western China, and incorporating larger, more diverse, and more multi-tiered nurse samples to verify and extend the conclusions of this study, thereby enhancing the generalizability and representativeness of the findings.

Second, the cross-sectional design captured the practice environment at only a single time point, precluding causal inference. Future longitudinal studies are needed to explore dynamic changes and causal mechanisms. Third, data were collected through nurse self-report, with both independent and dependent variables derived from the same source, potentially introducing common method bias. Future research could incorporate objective indicators or multi-source data for verification. Fourth, participation was voluntary; nurses who did not participate may differ systematically from participants, potentially introducing selection bias. Fifth, the explanatory power of the regression model was low (adjusted R^2^ = 0.060), suggesting the existence of important predictors not included in the model, such as workload, burnout, psychological safety, organizational culture, leadership style, and perceived organizational support. Future research should incorporate these variables to construct a more comprehensive predictive model. In summary, the findings of this study should be interpreted cautiously in light of the aforementioned limitations, and future research employing multicenter, longitudinal, and multi-source data designs is needed to further verify and deepen the relevant conclusions.

This study indicates that nurse practice environment scores are lower than those in maternal and child specialized hospitals in coastal regions, with department, gender, and position identified as influencing factors in the practice environment assessment. Accordingly, it is recommended that Chief Nursing Officers prioritize emergency department and ICU nurses by conducting targeted assessments, optimizing human resource allocation, and establishing flexible scheduling and staffing supplementation mechanisms based on workload and patient acuity. Concurrently, psychological support such as group stress reduction and individual counseling should be introduced, and career development pathways including specialty training, clinical advancement, and transfer support should be designed to reduce turnover intention and burnout. Attention should be paid to gender diversity within the nursing workforce by ensuring equity in scheduling, promotion, and training opportunities, and by encouraging male nurses to participate in fields such as obstetrics and pediatrics while providing developmental support. Furthermore, mechanisms for frontline nurse participation in management should be strengthened through the establishment of nurse councils and online suggestion platforms, safeguarding their voice in matters including scheduling, process improvement, and quality safety, with feedback and incentives provided for adopted suggestions to enhance professional belonging. These measures collectively aim to continuously optimize the practice environment and promote nursing workforce stability and improved nursing quality.

## Data Availability

The original contributions presented in the study are included in the article/Supplementary Material, further inquiries can be directed to the corresponding author.

## References

[1] National Nursing Quality Control Center. 2020 National Report on Healthcare Quality and Safety: Nursing Volume. Beijing: Science and Technology Literature Publishing House (2022).

[2] Zhang XM Ma XL Liu YJ Li ZX. Investigation on nursing practice environment in tertiary hospitals in shandong province. Chin J Nurs. (2020) 55(11):1680–4. 10.3761/j.issn.0254-1769.2020.11.013

[3] Yan W Chen X Xiao D Wang H Du X Li L. Patient mistreatment, social sharing of negative events and emotional exhaustion among Chinese nurses: the combined moderating effect of organizational support and trait resilience. BMC Nurs. (2024) 23(1):260. 10.1186/s12912-024-01924-x 38649852 PMC11036568

[4] Brassington S Thomas-Hawkins C Johansen ML De Cordova PB Cimiotti JP Zha P. Impact of registered nurse staffing, workload, and the practice environment on burnout in emergency nurses. J Emerg Nurs. (2025) 51(4):684–91. 10.1016/j.jen.2025.01.007 40057897

[5] Jennings R Outhwaite I Granek I Haq F. The effects of the physical and professional workplace environments on the well-being of nursing staff. Am J Nurs. (2025) 125(3):30–9. 10.1097/AJN.0000000000000033 39972585 PMC12856750

[6] Zeng Y Zhao X Xie Z Lin X Qi M Li P. Prediction of job burnout in nurses based on the job demands-resources model: an explainable machine learning approach. J Adv Nurs. (2026) 82(3):2232–44. 10.1111/jan.17071 40459061

[7] Read E Metersky K Matthews L MacLean R. Understanding the work life of clinical nurse educators in Canadian hospitals through the job demands-resources model: a descriptive qualitative study. Int J Nurs Stud. (2026) 173:105249. 10.1016/j.ijnurstu.2025.105249 41138663

[8] Wang X Liu M Xu T Wang K Huang L Zhang X. New nurses’ practice environment, job stress, and patient safety attitudes: a cross-sectional study based on the job demands-resources model. BMC Nurs. (2024) 23(1):473. 10.1186/s12912-024-02135-0 38997677 PMC11241995

[9] Eisenberger R Huntington R Hutchison S Sowa D. Perceived organizational support. J Appl Psychol. (1986) 71(3):500–7. 10.1037/0021-9010.71.3.500

[10] Zheng J Feng S Gao R Gong X Ji X Li Y. The relationship between organizational support, professional quality of life, decent work, and professional well-being among nurses: a cross-sectional study. BMC Nurs. (2024) 23(1):425. 10.1186/s12912-024-02114-5 38918776 PMC11197337

[11] Batino M Fiorini J Zaghini F Biagioli V Frigerio S Sili A. The relationship between nursing work environment and breastfeeding prevalence during child hospitalization: a multicenter study. Healthcare (Basel). (2024) 12(24):2574. 10.3390/healthcare12242574 39766001 PMC11728232

[12] Wu F Wang X Chen SC Li H Xie H. Nurses’ adverse event reporting attitudes and related factors: a cross-sectional study in maternal and child specialized hospitals in China. Front Public Health. (2024) 12:1357924. 10.3389/fpubh.2024.1434387 PMC11659206 39712313

[13] Li Y Zheng X Yang Z Yan W Li Q Liu Y. The mediating effect of job burnout on the relationship between practice environment and workplace deviance behavior of nurses in China: a cross-sectional study. BMC Nurs. (2025) 24(1):19. 10.1186/s12912-024-02663-9 39773632 PMC11705975

[14] Labrague LJ Al Sabei S AbuAlRub R Burney I Al Rawajfah O. The role of nurses’ adherence to clinical safety guidelines in linking nurse practice environment to missed nursing care. J Nurs Scholarsh. (2025) 57(2):354–62. 10.1111/jnu.13017 39160684

[15] Norful AA Brewer KC Cahir KM Dierkes AM. The impact of the nurse practice environment, workload, and professional support on job outcomes and standards of care at primary health care clinics in South Africa: a structural equation model approach. Int J Nurs Stud Adv. (2024) 6:100187. 10.1016/j.ijnsa.2024.100241 39351496 PMC11440313

[16] Zhang HY Wu ZJ Liang JQ Shang WH Jian WY Yo L. Revision of the nursing practice environment scale and evaluation of its reliability and validity. Chin Nurs Manage. (2019) 19(3):388–93. 10.3969/j.issn.1672-1756.2019.03.013

[17] Wang TM Liu Y Li FQ Li JX. Current status and influencing factors of nursing practice environment in tertiary grade A hospitals in sichuan province. Chin J Mod Nurs. (2022) 28(22):2963–8. 10.3760/cma.j.cn115682-20211103-04954

[18] Zheng YQ Tang GY. Practice environment and turnover intention among nurses in a tertiary pediatric hospital. Int J Nurs. (2023) 42(6):985–90. 10.3760./cma.j.cn.221370-20210713-00243

[19] Laschinger HK Finegan J Shamian J Wilk P. Impact of structural and psychological empowerment on job strain in nursing work settings. J Nurs Adm. (2001) 31(5):260–72. 10.1097/00005110-200105000-00006 11388162

[20] Lee SE Lee JW. Effects of hierarchical unit culture and power distance orientation on nurses’ silence behavior: the roles of perceived futility and hospital management support for patient safety. J Nurs Manag. (2024) 2024:6564570. 10.1155/jonm/6564570 40224770 PMC11925317

[21] Mauricio K Bulger AL Jurgens CY Ruscitti B Gregory KE Bowser D. Nurse practice environment, job satisfaction, and turnover and patient falls. JAMA Netw Open. (2026) 9(7):e2112345. 10.1001/jamanetworkopen.2025.51223 PMC12750249 41632167

[22] de Lima Trindade L Guerreiro BC De Oliveira SMS Ventura-Silva JMA Rostirolla L Zuge SS. Evaluation of professional nursing practice environments in primary health care. Front Public Health. (2025) 12:1477067. 10.3389/fpubh.2024.1477067 39911779 PMC11797917

[23] Gadolin C Skyvell Nilsson M Ros A Törner M. Preconditions for nurses’ perceived organizational support in healthcare: a qualitative explorative study. J Health Organ Manag. (2021) 35(9):281–97. 10.1108/JHOM-03-2020-0091 34523305 PMC9136871

[24] Polat Ş Terzi B. Relationships between perceived support types and the job satisfaction levels of nurses. Perspect Psychiatr Care. (2021) 57(3):1202–11. 10.1111/ppc.12675 33145771

[25] Cañadas-de la Fuente GA Albendín-García L R Cañadas G San Luis-Costas C Ortega-Campos E De La Fuente-Solana EI. Nurse burnout in critical care units and emergency departments: intensity and associated factors. Emergencias. (2018) 30(5):328–31. 30260117

[26] Collado-González B Urtubia-Herrera V Navarta-Sánchez MV Sanz-Garcia A Palmar-Santos AM; Paediatric Emergency Safety Culture Group. Safety culture in pediatric emergency departments: a cross-sectional study among healthcare professionals. BMJ Open. (2025) 15(3):e081234. 10.1136/bmjopen-2025-105434 PMC12542550 41120138

[27] Spreitzer GM. Psychological empowerment in the workplace: dimensions, measurement, and validation. Acad Manage J. (1995) 38(5):1442–65. 10.2307/256865

[28] Lim E Zhang M Higham K Donnelly F. Factors affecting the mental health and wellbeing of men in nursing: a systematic review and narrative synthesis. Int J Nurs Pract. (2026) 32(3):e70153. 10.1111/ijn.70153 42108413 PMC13158322

[29] Göktepe N Sarıköse S. Same place but different experience: a qualitative study on gender and the nursing work environment. J Nurs Manag. (2022) 30(7):3227–35. 10.1111/jonm.13748 35895493

[30] Zhu W Li Z Jiang L Han Y Jiang Y. Gender bias, psychological well-being, and professional adaptation among male nurses: a scoping review. Healthcare (Basel). (2026) 14(14):2095. 10.3390/healthcare14142095 42512611 PMC13410122

[31] He J Gao J Shi LY. Survey and influencing factors analysis of nursing practice environment among 5,017 nurses from 2017 to 2019. J Xinjiang Med Univ. (2021) 44(2):244–7. 10.3639/j.issn.1009-5551.2021.02.023

[32] Hamdan M Jaafar AH. The impact of shared governance model’s implementation on professional governance perceptions of nurses in Saudi Arabia: a randomised controlled trial. J Nurs Manag. (2024) 2024:1–8. 10.1155/2024/7263656 PMC11918916 40224824

[33] Jaber A Ta'an WF Aldalaykeh MK Al-Shannaq YM Oweidat IA Mukattash TL. The perception of shared governance and engagement in decision-making among nurses. Nurs Forum. (2022) 57(6):1169–75. 10.1111/nuf.12817 36315058

[34] Kiptulon EK Elmadani M Limungi GM Simon K Tóth L Horvath E. Transforming nursing work environments: the impact of organizational culture on work-related stress among nurses: a systematic review. BMC Health Serv Res. (2024) 24(1):1526. 10.1186/s12913-024-12003-x 39623348 PMC11613752

[35] Bakker AB Demerouti E. Job demands-resources theory: taking stock and looking forward. J Occup Health Psychol. (2017) 22(3):273–85. 10.1037/ocp0000056 27732008

